# Supplementary material for: The Influence of Alcohol Consumption, Depressive Symptoms and Sleep Duration on Cognition: Results from the China Health and Retirement Longitudinal Study
Source: Int J Environ Res Public Health. 2022 Oct 1;19(19):12574. doi: 10.3390/ijerph191912574 (PMC9566793; doi:10.3390/ijerph191912574)
Supplement: Supplementary file 1 [file ijerph-19-12574-s001.zip › ijerph-1942794-supplementary.pdf]

contents

Table S1 Sensitive Analyses for Baseline Characteristics of the Study Population (Totals and Stratified According to Sleep Duration, and Alcohol Consumption).

Table S2 Sensitive Analyses for Cross-Sectional Association Between Sleep Duration per Night, Alcohol Consumption (time per month), covariates, and Global Cognitive Function at Baseline Using Generalized Linear Model

Table S3 Sensitivity Analyses for Mean Difference in Rate of Change in Global Cognitive Decline During Follow-up Using Generalized Estimating Equation

Figure S1 Sensitivity Analyses for the Trajectory of Late Life Cognitive Decline with Age.

Table S1. Sensitive Analyses for Baseline Characteristics of the Study Population (Totals and Stratified According to Sleep Duration, and Alcohol Consumption).

	All	Sleep duration (hours per night) (n=10248)				Alcohol Consumption (time per month) (n=10272)			
		≤6h	7-8h	≥9h	***p* value**	Non-drinkers	<=1	>1	***p* value**
N (%)	11204	5055(49.3)	4327(42.2)	866(8.5)		6589(64.1)	852(8.3)	2831(27.6)	
Age (years), mean (SD)	59.02(10.16)	59.03(10.09)	59.07(10.21)	58.71(9.86)	**0.506**	58.98(10.09)	59.18(10.07)	59.06(10.19)	**0.708**
Gender(women), n (%)	5818(51.9)	2627(52.0)	2207(51.0)	473(54.7)	**0.136**	3423(52.0)	431(50.6)	1472(52.0)	**0.730**
Education level, n (%)					**0.253**				**0.265**
No formal education or illiterate	3030(27.1)	1371(27.2)	1132(26.2)	261(30.3)		1735(26.4)	238(28.0)	808(28.6)	
Did not finish primary school	1960(17.6)	895(17.8)	730(16.9)	148(17.2)		1143(17.4)	156(18.4)	503(17.8)	
Finished middle school	5865(52.5)	2637(52.3)	2321(53.8)	434(50.4)		3499(53.3)	430(50.6)	1446(51.3)	
Higher and further education	266(2.4)	119(2.4)	110(2.6)	16(1.9)		163(2.5)	22(2.6)	53(1.9)	
Others	46(0.4)	18(0.4)	20(0.5)	2(0.2)		26(0.4)	4(0.5)	11(0.4)	

Living with a partner, n (%)	8980(80.1)	4056(80.3)	3449(79.9)	698(80.9)	**0.825**	5295(80.5)	680(79.8)	2267(80.2)	**0.069**
Cigarette consumption, n (%)	4451(39.7)	1962(38.8)	1726(39.9)	316(36.5)	**0.148**	2609(39.7)	339(39.9)	1155(40.9)	**0.540**
Blood pressure, mean (SD), mm Hg									
Systolic	130.53(21.75)	130.20(21.61)	130.98(22.08)	131.29(21.99)	**0.290**	130.67(21.89)	132.14(23.31)	130.02(21.23)	**0.123**
Diastolic	75.86(12.21)	75.78(12.19)	76.01(12.44)	76.03(11.92)	**0.781**	75.89(12.19)	76.30(13.82)	75.80(11.96)	**0.508**
Body mass index (kg/m2)	23.46(3.98)	23.46(4.01)	23.45(4.00)	23.44(3.96)	**0.845**	23.39(3.97)	23.46(3.75)	23.61(4.14)	**0.166**
Cognitive function domain scores (z-scores), mean (SD)									
The memory score	0.08(1.01)	0.09(1.00)	0.15(1.01)	0.14(0.98)	**0.020**	0.11(1.03)	0.26(0.99)	0.12(0.97)	**0.001**
The orientation test score	0.09(0.96)	0.13(0.95)	0.16(0.92)	0.11(0.93)	**0.117**	0.10(0.95)	0.28(0.84)	0.18(0.90)	**<0.001**
The executive score	0.03(1.04)	0.34(0.91)	0.38(0.88)	0.34(0.90)	**0.091**	0.27(0.92)	0.50(0.83)	0.51(0.83)	**<0.001**
The total z scores	0.11(0.99)	0.08(1.00)	0.15(0.98)	0.12(0.96)	**0.023**	0.05(1.01)	0.30(0.93)	0.21(0.93)	**<0.001**
Hypertension, n (%)	2728(24.3)	1282(25.5)	1022(23.7)	208(24.0)	**0.114**	1615(24.7)	211(24.9)	683(24.3)	**0.924**
Diabetes, n (%)	621(5.5)	305(6.1)	236(5.5)	36(4.2)	**0.065**	379(5.8)	62(7.4)	132(4.7)	**0.008**
Dyslipidemia, n (%)	1026(9.2)	494(10.0)	396(9.3)	69(8.1)	**0.185**	623(9.7)	86(10.3)	247(8.9)	**0.369**
Coronary heart disease, n (%)	1300(11.6)	696(13.9)	444(10.3)	83(9.7)	**<0.001**	761(11.6)	98(11.6)	335(11.9)	**0.931**
Stroke, n (%)	212(1.9)	108(2.1)	67(1.6)	19(2.2)	**0.091**	127(1.9)	11(1.3)	59(2.1)	**0.338**
Chronic lung disease,	1050(9.4)	576(11.4)	335(7.8)	69(8.0)	**<0.001**	593(9.1)	92(10.8)	269(9.6)	**0.219**

n (%)									
Asthma, n (%)	375(3.3)	211(4.2)	108(2.5)	30(3.5)	**<0.001**	213(3.2)	28(3.3)	103(3.7)	**0.605**

Table S2. Sensitive Analyses for Cross-Sectional Association Between Sleep Duration per Night, Alcohol Consumption (time per month), covariates, and Global Cognitive Function at Baseline Using Generalized Linear Model

	**B,95%CI, P Value**							
	**The memory score**		**The orientation test score**		**The executive score**		**The total z scores**	
	Model1[a] (n=7414)	Model2[b] (n=5848)	Model1[a] (n=7725)	Model2[b] (n=6130)	Model1[a] (n=9332)	Model2[b] (n=7439)	Model1[a] (n=6994)	Model2[b] (n=5517)
Sleep duration (hours per 24h period)								
⩽6h	-0.140	-0.147	-0.055	-0.034	-0.049	-0.076	-0.119	-0.127
	-0.233 to -0.047	-0.252 to -0.041	-0.141 to 0.030	-0.130 to 0.061	-0.133 to 0.035	-0.171 to 0.018	-0.211 to -0.028	-0.230 to -0.0024
***P* value**	**0.003**	**0.006**	**0.202**	**0.480**	**0.249**	**0.113**	**0.011**	**0.016**
7-8h	REF	REF	REF	REF	REF	REF	REF	REF
⩾9h	-0.011	0.029	-0.105	-0.051	0.007	0.037	-0.004	0.034
	-0.182 to 0.159	-0.168 to 0.226	-0.261 to 0.051	-0.228 to 0.127	-0.148 to 0.162	-0.141 to 0.214	-0.173 to 0.165	-0.160 to 0.228

***P* value**	**0.896**	**0.771**	**0.185**	**0.577**	**0.928**	**0.687**	**0.962**	**0.731**
Whether exposed to alcohol (time per month)								
Non-drinkers	REF	REF	REF	REF	REF	REF	REF	REF
<=1	0.356	0.248	0.165	0.161	0.196	0.026	0.401	0.279
	0.046 to 0.666	-0.131 to 0.628	-0.110 to 0.441	-0.167 to 0.489	-0.077 to 0.469	-0.293 to 0.345	0.097 to 0.706	-0.087 to 0.644
***P* value**	**0.025**	**0.199**	**0.240**	**0.337**	**0.160**	**0.873**	**0.010**	**0.135**
>1	0.107	0.130	0.052	0.067	0.332	0.335	0.243	0.261
	-0.079 to 0.292	-0.085 to 0.344	-0.118 to 0.222	-0.126 to 0.260	0.165 to 0.500	0.144 to 0.526	0.058 to 0.427	0.049 to 0.472
***P* value**	**0.260**	**0.236**	**0.548**	**0.494**	**<0.001**	**0.001**	**0.010**	**0.016**
Age(years)	0.002	0.001	0.000	-0.001	0.001	0.001	0.002	0.002
	-0.001 to 0.004	-0.001 to 0.004	-0.002 to 0.002	-0.003 to 0.002	-0.001 to 0.004	-0.001 to 0.004	-0.001 to 0.004	-0.001 to 0.004
***P* value**	**0.163**	**0.317**	**0.973**	**0.570**	**0.206**	**0.290**	**0.157**	**0.282**
Gender	0.019	0.016	0.008	0.001	0.014	-0.008	0.023	0.014
	-0.031 to 0.068	-0.041 to 0.072	-0.037 to 0.054	-0.050 to 0.052	-0.030 to 0.059	-0.058 to 0.042	-0.026 to 0.073	-0.042 to 0.069
***P* value**	**0.460**	**0.586**	**0.725**	**0.966**	**0.528**	**0.756**	**0.352**	**0.629**

Education level	0.021	0.017	0.014	0.012	0.018	0.015	0.025	0.024
	0.008 to 0.033	0.002 to 0.032	0.002 to 0.026	-0.001 to 0.025	0.006 to 0.030	0.002 to 0.028	0.012 to 0.037	0.010 to 0.039
***P* value**	**0.002**	**0.023**	**0.024**	**0.077**	**0.003**	**0.025**	**0.001**	**0.001**
Marriage	0.002	0.007	-0.013	-0.007	-0.002	0.001	0.002	0.008
	-0.017 to 0.021	-0.014 to 0.028	-0.030 to 0.004	-0.026 to 0.012	-0.019 to 0.015	-0.018 to 0.020	-0.017 to 0.021	-0.013 to 0.029
***P* value**	**0.825**	**0.519**	**0.138**	**0.473**	**0.843**	**0.898**	**0.818**	**0.457**
Blood pressure								
Systolic	NA	-0.002	NA	0.000	NA	-0.001	NA	-0.001
		-0.005 to 0.002		-0.002 to 0.001		-0.002 to 0.001		-0.004 to 0.002
***P* value**		**0.309**		**0.851**		**0.446**		**0.537**
Diastolic	NA	0.000	NA	-0.001	NA	-0.001	NA	0.000
		-0.002 to 0.002		-0.004 to 0.002		-0.004 to 0.002		-0.002 to 0.002
***P* value**		**0.864**		**0.436**		**0.403**		**0.938**
Body mass index (kg/m2)	NA	0.000	NA	0.000	NA	-0.004	NA	-0.004
		-0.007 to 0.007		-0.006 to 0.006		-0.010 to 0.002		-0.010 to 0.003
***P* value**		**0.990**		**0.881**		**0.171**		**0.291**

Cigarette consumption		0.027		0.031		0.011		0.050
	NA	-0.027 to 0.081	NA	-0.018 to 0.080	NA	-0.038 to 0.059	NA	-0.003 to 0.103
***P* value**		**0.326**		**0.215**		**0.668**		**0.067**
Hypertension		0.075		-0.008		-0.019		0.031
	NA	0.009 to 0.140	NA	-0.067 to 0.052	NA	-0.078 to 0.039	NA	-0.034 to 0.096
***P* value**		**0.026**		**0.799**		**0.520**		**0.354**
Diabetes		-0.078		-0.014		0.009		-0.077
	NA	-0.200 to 0.044	NA	-0.125 to 0.097	NA	-0.100 to 0.118	NA	-0.199 to 0.045
***P* value**		**0.211**		**0.802**		**0.871**		**0.217**
Dyslipidemia		0.032		0.016		0.028		0.042
	NA	-0.067 to 0.130	NA	-0.073 to 0.105	NA	-0.059 to 0.115	NA	-0.055 to 0.138
***P* value**		**0.527**		**0.727**		**0.525**		**0.397**

Coronary heart disease	NA	-0.035<br>-0.120 to 0.050	NA	-0.019<br>-0.097 to 0.059	NA	0.006<br>-0.071 to 0.082	NA	-0.045<br>-0.129 to 0.040
***P* value**		**0.424**		**0.636**		**0.887**		**0.300**
Stroke	NA	-0.030<br>-0.226 to 0.166	NA	0.012<br>-0.168 to 0.193	NA	-0.059<br>-0.236 to 0.117	NA	-0.016<br>-0.211 to 0.180
***P* value**		**0.762**		**0.895**		**0.509**		**0.875**
Chronic lung disease	NA	0.002<br>-0.092 to 0.096	NA	-0.013<br>-0.099 to 0.073	NA	0.052<br>-0.032 to 0.136	NA	0.014<br>-0.080 to 0.108
***P* value**		**0.970**		**0.765**		**0.222**		**0.777**
Asthma	NA	0.155<br>0.004 to 0.306	NA	0.098<br>-0.039 to 0.235	NA	-0.034<br>-0.170 to 0.103	NA	0.136<br>-0.012 to 0.285
***P* value**		**0.044**		**0.161**		**0.628**		**0.072**

Table S3. Sensitivity Analyses for Mean Difference in Rate of Change in Global Cognitive Decline During Follow-up Using Generalized Estimating Equation

	β (95% CI) [a]	p Value
Sleep duration (hours per 24h period)		
⩽6h	-0.121 (-0.192 to -0.051)	<0.05
7-8h	REF	REF
⩾9h	0.007 (-0.126 to 0.139)	0.923
Whether exposed to alcohol (time per month)		
No	REF	REF
<=1	0.186 (-0.056 to 0.427)	0.133
>1	0.117 (-0.027 to 0.261)	0.112
Sleep duration * Alcohol consumption		0.057
Age (years)	-0.001 (-0.003 to 0.001)	0.213
Gender	.026 (-0.012 to 00.063)	0.183
Education level	0.012 (0.002 to 0.022)	<0.05
Marriage	0.008 (-0.006 to 0.022)	0.250
Blood pressure		

Systolic	0.001 (-0.001 to 0.002)	0.273
Diastolic	-0.001 (-0.003 to 0.001)	0.303
Body mass index (kg/m2)	-0.001 (-0.005 to 0.004)	0.721
Cigarette consumption	0.033 (-0.003 to 0.070)	0.071
Hypertension	0.031 (-0.013 to 0.075)	0.162
Diabetes	-0.089 (-0.171 to -0.006)	<0.05
Dyslipidemia	-0.059 (-0.124 to 0.006)	0.077
Coronary heart disease	-0.035 (-0.092 to 0.023)	0.234
Stroke	-0.017 (-0.149 to 0.115)	0.802
Chronic lung disease	0.027 (-0.037 to 0.090)	0.409
Asthma	0.032 (-0.068 to 0.133)	0.529

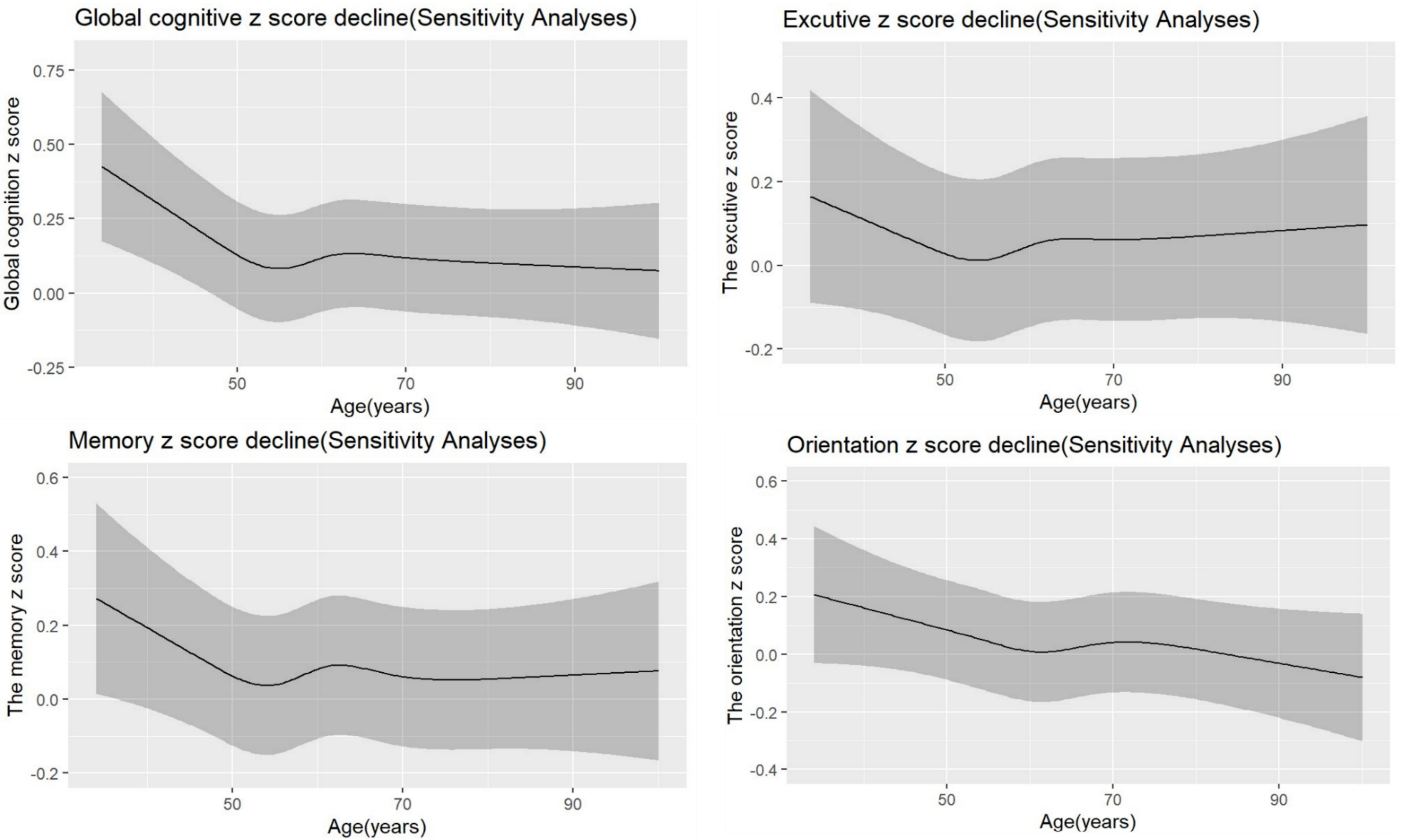

Figure S1 Sensitivity Analyses for The Trajectory of Late Life Cognitive Decline with Age.
